# Supplementary material for: Experimental Demonstration on Quantum Sensitivity to Available Information in Decision Making
Source: Sci Rep. 2019 Jan 24;9:681. doi: 10.1038/s41598-018-36945-5 (PMC6346084; doi:10.1038/s41598-018-36945-5)
Supplement: Supplementary file 1 — Supplementary Material for [file 41598_2018_36945_MOESM1_ESM.pdf]

Supplementary Material for "Experimental Demonstration on Quantum Sensitivity to Available Information in Decision Making"

Joong-Sung Lee,[1] Jeongho Bang,[2,3,*] Jinhyoung Lee,[1,†] and Kwang-Geol Lee[1,‡]

[1]*Department of Physics, Hanyang University, Seoul 04763, Korea*

[2]*School of Computational Sciences, Korea Institute for Advanced Study, Seoul 02455, Korea*

[3]*Institute of Theoretical Physics and Astrophysics, University of Gdańsk, 80-952 Gdańsk, Poland*

Supplementary Material for "Experimental Demonstration on Quantum Sensitivity to Available Information in Decision Making."

S1. THE THEORETICAL ANALYSIS OF BOB'S AVERAGE PAYOFF (SCORE)

A. The operations u_j ($j = 0, 1$) in the classical and quantum decision-making process

In our study, Bob's decision-making (DM) process can be described as a function $f : x_\kappa \to m_\kappa$ ($\kappa = 0, 1$). Here, the measurement outcome m_κ is supposed to be the outcomes of Bob's decision, i.e., $m_\kappa \to y_\kappa$, as described in the main manuscript. The function f is defined with the two operations u_0 and u_1 in the ancillary system. More specifically, it implements four possible functions f depending on the pair (u_0, u_1), such that

$$\begin{aligned}
&[\tau.1] \quad (\mathbb{1}, \mathbb{1}) \leftrightarrow f_{\tau=1}(x_\kappa) = 0, \\
&[\tau.2] \quad (\mathbb{1}, X) \leftrightarrow f_{\tau=2}(x_\kappa) = x_\kappa, \\
&[\tau.3] \quad (X, \mathbb{1}) \leftrightarrow f_{\tau=3}(x_\kappa) = 1, \\
&[\tau.4] \quad (X, X) \leftrightarrow f_{\tau=4}(x_\kappa) = 1 \oplus x_\kappa,
\end{aligned} \tag{S1}$$

where $\mathbb{1}$ and X denote the identity and logical-not operations, respectively. Here, we set $\alpha = 0$ for simplicity (see Table in Fig. 2 of the main manuscript). Then, we recall the classical and quantum versions of DM process. As described in our main text, cDM is defined with the classical elements of the ancillary system; the ancilla input $\alpha = 0$ is a binary number, and the operations u_j are applied randomly (either to be $\mathbb{1}$ or to be X) based on the preferences $P(u_j \to \mathbb{1})$ and $P(u_j \to X)$

*Electronic address: jbang@kias.re.kr

†Electronic address: hyoung@hanyang.ac.kr

‡Electronic address: kglee@hanyang.ac.kr

$(j = 0, 1)$. Thus, the probabilistic application of u_j is represented by a stochastic evolution matrix,

$$\begin{pmatrix} P(u_j \to \mathbb{1}) & P(u_j \to X) \\ P(u_j \to X) & P(u_j \to \mathbb{1}) \end{pmatrix}. \tag{S2}$$

On the other hand, the qDM is defined with the quantum input $|\alpha\rangle = |0\rangle$ and the application of u_j is represented by a unitary matrix,

$$\begin{pmatrix} \sqrt{P(u_j \to \mathbb{1})} & e^{i\phi_j}\sqrt{P(u_j \to X)} \\ e^{-i\phi_j}\sqrt{P(u_j \to X)} & -\sqrt{P(u_j \to \mathbb{1})} \end{pmatrix}. \tag{S3}$$

which inherently involves (quantum) probabilistic properties. Here, note that the additional degree of freedom, i.e., the quantum phase ϕ_j $(j = 0, 1)$, is introduced to faithfully deal with the quantum superposition property.

B. The calculations of Bob's payoffs

One crucial task in game theory is to characterize a function \$, which determines the average payoffs of the players over the number of games:

$$\$: S \times H \to \Xi^{(i)} \in \mathbb{R}, \tag{S4}$$

where S and H denote the set of possible strategies and preferences, respectively. Here, $\Xi^{(i)}$ is the average payoff of the i-th player. In our game, Bob's average payoff Ξ can be written, explicitly, as

$$\Xi = \frac{1}{4}\sum_{\tau=1}^{4}\overline{\xi}_\tau \tag{S5}$$

where we assumed that Alice chooses her secret bits x_κ at random. The value $\overline{\xi}_\tau$ $(\tau = 1, 2, 3, 4)$ is defined as the payoff averaged for a specific cases of τ, defined in Eq. (S1), i.e.,

$$\overline{\xi}_\tau = \sum_{x_\kappa \in \{0,1\}} \frac{\xi}{2}\Big(P(x_\kappa = y_\kappa) - P(x_\kappa \neq y_\kappa)\Big), \tag{S6}$$

where the index τ specifies one of the cases [τ.1]-[τ.4]. Here, $P(x_\kappa = y_\kappa)$ and $P(x_\kappa \neq y_\kappa)$ are the probabilities that the outcome of Bob's decision is correct and incorrect for the given x_κ, respectively. For later analysis, we rewrite Eq. (S6), for each τ, as below

$$\begin{aligned}
\overline{\xi}_{\tau=1} &= \frac{\xi}{2}\Big(P(y_0 = 0) + P(y_1 = 0) - P(y_0 = 1) - P(y_1 = 1)\Big), \\
\overline{\xi}_{\tau=2} &= \frac{\xi}{2}\Big(P(y_0 = 0) + P(y_1 = 1) - P(y_0 = 1) - P(y_1 = 0)\Big), \\
\overline{\xi}_{\tau=3} &= \frac{\xi}{2}\Big(P(y_0 = 1) + P(y_1 = 1) - P(y_0 = 0) - P(y_1 = 0)\Big), \\
\overline{\xi}_{\tau=4} &= \frac{\xi}{2}\Big(P(y_0 = 1) + P(y_1 = 0) - P(y_0 = 0) - P(y_1 = 1)\Big),
\end{aligned} \tag{S7}$$

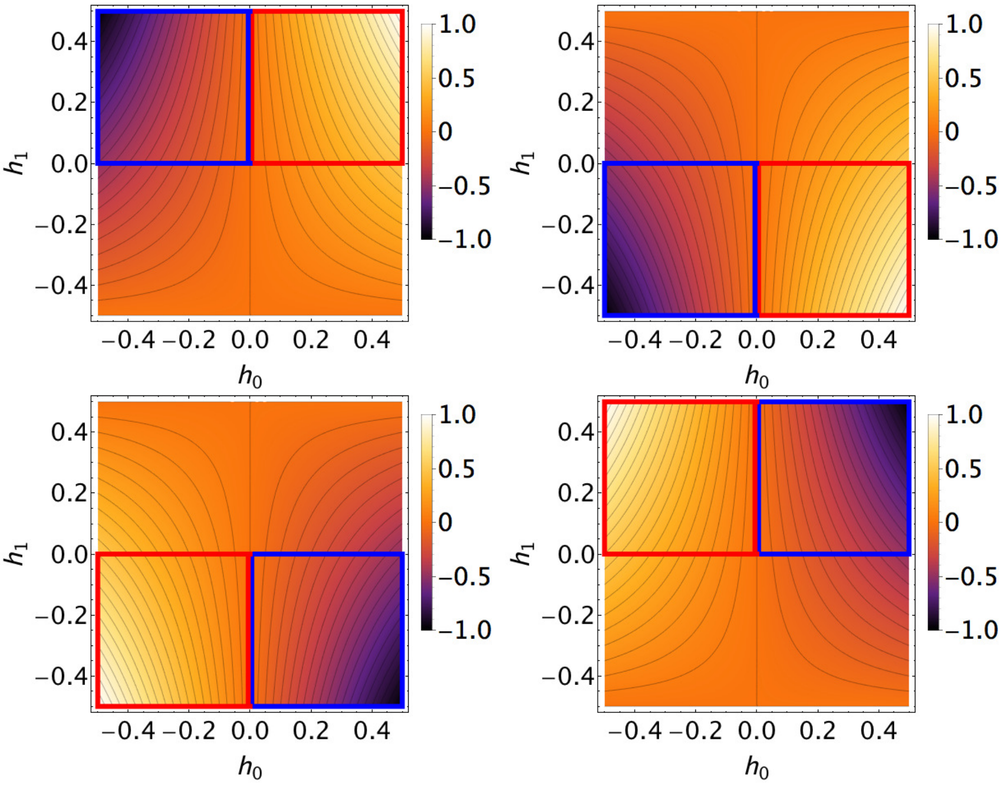

FIG. S1: **Bob's average payoffs in cDM.** We depict the theoretically expected $\overline{\xi}_{\tau,C}$ averaged for a specific set of $x_\tau \in \{0,1\}$: (top-left) [τ.1], (top-right) [τ.2], (bottom-left) [τ.3], and (bottom-right) [τ.4]. We specify the regions of the good hints (red-line box) and the poor hints (blue-line box) (see, also, Fig. 4 in our main text).

where $P(y_\kappa = m_\kappa)$ is the probability of choosing the final strategy $y_\kappa = m_\kappa$ ($m_\kappa = 0, 1$) in our DM algorithm, described in Fig. 2 in the main manuscript.

1) Analysis of cDM. – Now, we calculate Bob's average payoff Ξ_C achievable from cDM. To do this, let us first write the classical probabilities $P_C(y_\kappa = m_\kappa)$ in Eq. (S7) in terms of the DM preferences casted in Eq. (S2), such that

$$\begin{aligned}
P_C(y_0=0) &= P(u_0 \to \mathbb{1}) = \frac{1}{2} + h_0, \\
P_C(y_0=1) &= P(u_0 \to X) = \frac{1}{2} - h_0, \\
P_C(y_1=0) &= P(u_0 \to \mathbb{1})P(u_1 \to \mathbb{1}) + P(u_0 \to X)P(u_1 \to X) = \frac{1}{2} + 2h_0h_1, \\
P_C(y_1=1) &= P(u_0 \to \mathbb{1})P(u_1 \to X) + P(u_0 \to X)P(u_1 \to \mathbb{1}) = \frac{1}{2} - 2h_0h_1. \qquad \text{(S8)}
\end{aligned}$$

Then, we can write $\overline{\xi}_{\tau,C}$ for cDM, using Eqs. (S6)-(S8), as below.

$$\begin{aligned}\overline{\xi}_{\tau=1,C} &= h_0 + 2h_0h_1,\\ \overline{\xi}_{\tau=2,C} &= h_0 - 2h_0h_1,\\ \overline{\xi}_{\tau=3,C} &= -h_0 + 2h_0h_1,\\ \overline{\xi}_{\tau=4,C} &= -h_0 - 2h_0h_1,\end{aligned} \tag{S9}$$

where the constant ξ is assumed to be 1 without loss of the generality. Here, it is obvious that if there is no bias among the preferences, i.e., no hints are provided as $h_0 = h_1 = 0$, then $\overline{\xi}_{\tau,C} = 0$ for all $\tau = 1, 2, 3, 4$. However, if Bob has non-zero hints $\mathbf{h} = (h_0, h_1)^T$, Bob can improve his winning average with good hint $\mathbf{h}$. Here, by "good" we mean that the directional conditions of $\mathbf{h}$ is appropriately assigned toward $(x_0, x_1)^T$. More specifically, Bob can have

$$\Xi_C = |h_0| + 2|h_0||h_1|. \tag{S10}$$

However, if the hint is poor, Bob may fail. In particular, we can imagine the worst case that any malicious hint misleads Bob, in which Bob will have the payoff

$$\Xi_C = -|h_0| - 2|h_0||h_1|. \tag{S11}$$

To see this clearly, we draw the graphs of $\overline{\xi}_{\tau,C}$ for $\tau = 1, 2, 3, 4$ based on the theoretical analysis (see Fig. S1). In each graph, we specify the regions of the good hints (red-line box) and the poor hints (blue-line box) in the space of $(h_0,\ h_1)$. This is well matched to our experimental results in Fig. 4 of the main manuscript.

2) Analysis of qDM. – Turning our analysis to the qDM, let us first write the quantum version of the probabilities $P_Q(y_\kappa = m_\kappa)$ as below

$$\begin{aligned}P_Q(y_0 = 0) &= |\langle m_0 = 0|\,\hat{u}_0\,|\alpha\rangle|^2 = P(u_0 \to \mathbb{1}) = \frac{1}{2} + h_0,\\ P_Q(y_0 = 1) &= |\langle m_0 = 1|\,\hat{u}_0\,|\alpha\rangle|^2 = P(u_0 \to X) = \frac{1}{2} - h_0,\\ P_Q(y_1 = 0) &= |\langle m_0 = 0|\,\hat{u}_1\hat{u}_0\,|\alpha\rangle|^2 = P(u_0 \to \mathbb{1})P(u_1 \to \mathbb{1}) + P(u_0 \to X)P(u_1 \to X)\\ &= \frac{1}{2} + 2h_0h_1 + \Gamma\cos(\pi\Delta),\\ P_Q(y_1 = 1) &= |\langle m_0 = 1|\,\hat{u}_1\hat{u}_0\,|\alpha\rangle|^2 = P(u_0 \to \mathbb{1})P(u_1 \to X) + P(u_0 \to X)P(u_1 \to \mathbb{1})\\ &= \frac{1}{2} - 2h_0h_1 - \Gamma\cos(\pi\Delta),\end{aligned} \tag{S12}$$

where $\Delta = |\phi_1 - \phi_0|$ and $\hat{u}_{0,1}$ denotes the unitary operation of $u_{0,1}$ in Eq. (S3). Here, Γ is given as [see Eq. (6) of the main text]

$$\Gamma = 2\sqrt{\left(\frac{1}{4} - |h_0|^2\right)\left(\frac{1}{4} - |h_1|^2\right)}. \tag{S13}$$

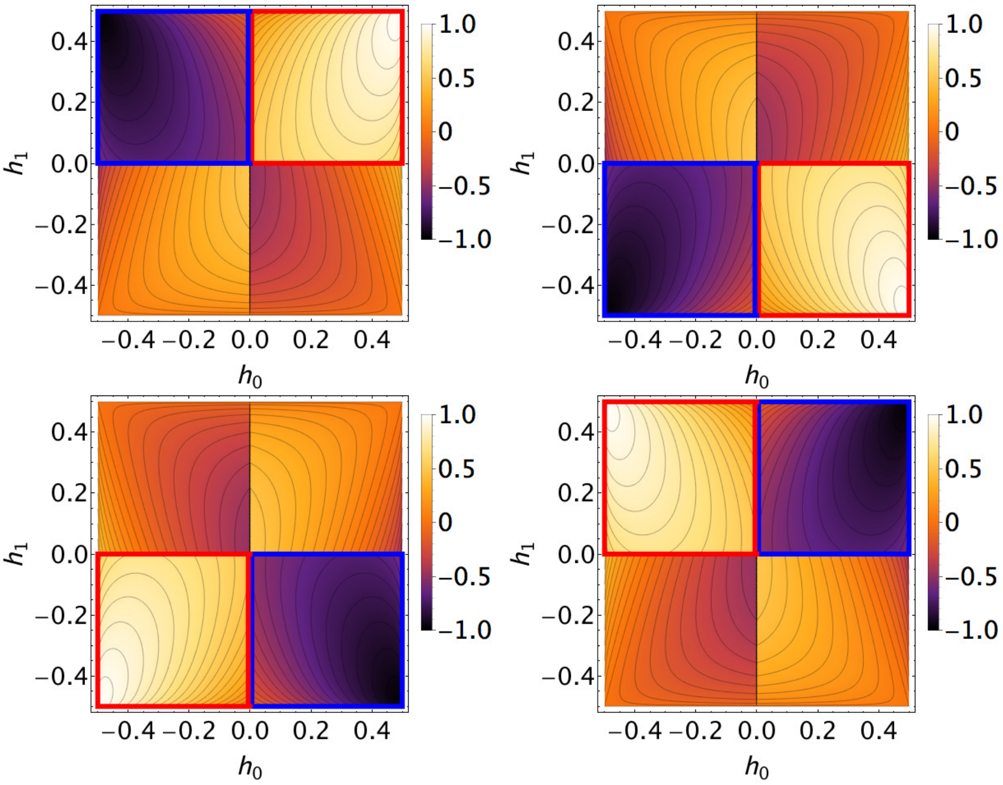

FIG. S2: **Bob's average payoffs in qDM.** We depict the theoretically expected $\overline{\xi}_{\tau,Q}$ for (top-left) [τ.1], (top-right) [τ.2], (bottom-left) [τ.3], and (bottom-right) [τ.4]. We also specify the regions of the good hints (red-line box) and the poor hints (blue-line box) (see, also, Fig. 4 in our main text).

Then, we can write $\xi_{\tau,Q}$, using Eq. (S6), Eq. (S7) and Eq. (S12), as

$$\begin{aligned}
\overline{\xi}_{\tau=1,Q} &= \overline{\xi}_{\tau=1,C} + \Gamma\cos(\pi\Delta), \\
\overline{\xi}_{\tau=2,Q} &= \overline{\xi}_{\tau=2,C} - \Gamma\cos(\pi\Delta), \\
\overline{\xi}_{\tau=3,Q} &= \overline{\xi}_{\tau=3,C} + \Gamma\cos(\pi\Delta), \\
\overline{\xi}_{\tau=4,Q} &= \overline{\xi}_{\tau=4,C} - \Gamma\cos(\pi\Delta),
\end{aligned} \tag{S14}$$

where ξ is also assumed to be 1 and Δ is determined by Eq. (4) in the main manuscript. Here, it is also true that Bob cannot improve his winning chance when $h_0 = h_1 = 0$; i.e, Bob has $\overline{\xi}_{\tau,Q} = 0$ for all $\tau = 1, 2, 3, 4$. However, it can be found from Eq. (S14) that the average payoff in the qDM can be higher than those in the cDM by Γ when provided by a proper value of Δ (good hint);

$$\Xi_Q = \Xi_C + \Gamma, \tag{S15}$$

as described also in the main text. However, there can also be malicious hinting, in which case Bob may fail, similarly to the classical case. From the same analysis as in the case of the cDM, we

can see that Bob's average Payoff can be decreased. Notably, in the worst case, such disadvantages can be maximized as

$$\Xi_Q = \Xi_C - \Gamma, \tag{S16}$$

This implies that the qDM can make the situation worse. To see this, let us see the theoretical graphs of $\overline{\xi}_{\tau,Q}$ in Fig. S2, where the regions of the good hints (red-line box) and the poor hints (blue-line box) are also specified.
